# Synthesis, characterization and anticancer activities of Zn^2+^, Cu^2+^, Co^2+^ and Ni^2+^ complexes involving chiral amino alcohols†

**DOI:** 10.1039/d2ra05576g

**Published:** 2022-11-09

**Authors:** Q. Umar, Y. H. Huang, A. Nazeer, H. Yin, J. C. Zhang, M. Luo, X. G. Meng

**Affiliations:** a Department of Chemistry and Chemical Engineering, Hefei University of Technology Hefei 23000 P.R. China luomei@pku.edu.cn mengxianggao@ccnu.edu.cn; b College of Chemistry, Central China Normal University Wuhan 430079 P.R. China

## Abstract

Seven new metal coordination complexes, [NiC_15_H_43_N_5_O_11_] (I), [Co_3_C_36_H_98_N_6_O_6_] (II), [CuC_14_H_32_N_2_O_6_] (III), [Cu_2_C_32_H_43_Cl_2_N_2_O_13_] (IV), [Zn_2_C_24_H_32_Cl_3_N_3_O_3_] (V), [Co_3_C_48_H_66_Cl_6_N_6_O_6_] (VI), and [Zn (C_18_H_45_N_3_O_3_] (VII), have been synthesized from some direct reactions of amino-alcoholic ligands with metal salts in anhydrous methanol or ethanol medium. All the crystals of these seven complexes are crystallized in the chiral space groups (*P*2_1_2_1_2_1_ for (I), (IV), (VI) and (VII); *P*2_1_ for (III) and (V); and C_2_ for (II), respectively). Their characteristic peaks were analyzed and assigned by FTIR, NMR, and UV–Vis and elemental analysis techniques. The anticancer activities of amino alcohol complexes (I)–(VII) showed cytotoxic effects against the human tumour cell line A549; among them, complex (V) showed the best activity with an IC_50_ value of 17.8. The higher biological activity should be related to its di-nuclear zinc(ii) unit in which one zinc is only four-coordinated by four small chloride anions.

## Abstract 2

A new type of coordination complexes related with the first transition metal and chiral amino alcohols can effectively fight against the human tumour cell line A549 with an IC_50_ value of 17.71 μM.

## Graphical abstract

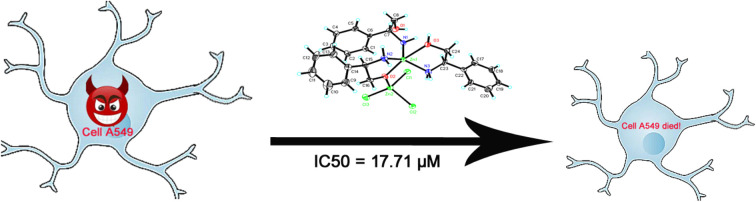

## Introduction

Cancer, as one of the worst lethal factors, is affecting human health all over the world.^1^ As a very cutting-edge subject, it is getting more and more attention from biologists, medical scientists and chemists.^2^ Especially, designing specific anticancer drug molecules based on a particular targeted cancer location is a major challenge for chemists. Although a large number of anti-cancer drug molecules have been designed so far, there are only a few of them that can be truly highly active during a treatment in clinical applications due to their side effects and drug resistance. Cisplatin, for example, is a molecule that has been recognized as having high therapeutic activity against cancer.^3–8^ There is no doubt that the discovery of cisplatin, *cis*-diamminedichloroplatinum(ii), represents one of the most significant events for cancer chemotherapy in the 20^th^ century.^9–12^ Besides cisplatin, several other platinum complexes (carboplatin, oxaliplatin, nedaplatin, and lobaplatin) have also been approved for current tumor therapy.^13^ Nedaplatin is another platinum derivative developed in Japan which is reported to cause much less nephrotoxicity than cisplatin.^13–15^ However, this potential drug molecule still needs more further experimental evidence before its real clinical practice application and efficacy recognized by researchers from all walks of life. It is precisely for this reason that chemists are constantly investing a lot of manpower and material resources to design a variety of small chemical molecules including organics, inorganic and metal complexes, hoping to be able to screen out some more artificial molecules with high cancer activities.

In more recent years, there have been still some other studies on the therapeutic applications using gold complexes as potential alternatives to the anticancer drug cisplatin in which the ligands are mainly related with some multi-dentate N-donor ligands.^16–18^ For instance, metal complexes based on ruthenium and iridium have been explored and they also show their respective different characteristics of biological activity in their anticancer activities research.^19–21^ However, the use of above mentioned nobel metals is still limited by its own shortcomings and limitations just like high preparation cost and poor compatibility of the human body. Recently, amino alcohols involved coordination compounds with the first transitional metal such as Co^2+^, Cu^2+^ and Zn^2+^ and have already been studied because of their good anticancer activities and low toxicity.^22–24^ What’ more, amino alcohol complexes can also play a key role in catalysis one hand and become a vital aspect of human life on the other hand. Most of the first-transition metal such as cobalt, copper and zinc complexes are all also very essential for living organisms. Aiming to find some new types of functional drug molecules, our research team have successfully synthesized and characterized a series of Cu^2+^, Zn^2+^, Co^2+^, and Ni^2+^ complexes by using a one-pot synthetic method. Additionally, the synthetic method has some advantages such as high yield, low toxicity, less smoke generation, and low cost, *etc.* The design strategy of metal coordination complexes are mainly based on the wide range of coordination numbers, inconstant geometries and available redox states.^8^

Through the one-pot synthetic method, we initially reported herein seven new complexes, [NiC_15_H_43_N_5_O_11_] (I), [Co_3_C_36_H_98_N_6_O_6_] (II), [CuC_14_H_32_N_2_O_6_] (III), [Cu_2_C_32_H_43_Cl_2_N_2_O_13_] (IV), [Zn_2_C_24_H_32_Cl_3_N_3_O_3_] (V), [Co_3_C_48_H_66_Cl_6_N_6_O_6_] (VI), and [ZnC_18_H_45_N_3_O_3_] (VII). Interestingly, these complexes demonstrated good cytotoxicity in lung cancer cells but negligible toxicity to normal cells.

## Experimental

### Materials and methods

d-Valinol, d-phenylglycenol, d-leucinol, Ni (NO_3_)_2_·6H_2_O, Co(OAc)_2_.4H_2_O, Cu (OAc) _2_.H_2_O, CuCl_2_·2H_2_O, CoCl_2_·6H_2_O, ZnCl_2_, and Cu(ClO_4_)_2_·6H_2_O were purchased from Acros. ^1^HNMR spectra were obtained using a Bruker AM-300 spectrometer. ^1^H and ^13^C NMR spectra were recorded using Bruker AM-500 and Bruker AM-600 spectrometers. Chemical shifts were reported in ppm (*δ*) with the solvent relative to tetramethylsilane (TMS) used as the internal standard (residual CHCl_3_, *δ*_H_ 7.26 ppm; CDCl_3_, *C*, 77 ppm). The following abbreviations were used to designate multiplicities: s = singlet, d = doublet, t = triplet, m = multiplet. Infrared spectra were recorded on a Mattson Galaxy Series FTIR 3000 spectrometer; peaks are reported in cm^−1^. Elemental analyses were performed on an Elemental Analyser AE-3000. The crystal structure was determined by a Gemini S Ultra diffractometer.

### Cytotoxicity assay

The human tumor cell line against A549 (lung cancer) was used in the cytotoxic assay. These cell lines were obtained from ATCC (Manassas, VA, USA). Cells were cultured in RMPI-1640 or DMEM (Biological Industries, Kibbutz Beit Haemek, and Israel) supplemented with 10% foetal bovine serum (Biological Industries) at 37 °C in a humidified atmosphere with 5% CO_2_. The cytotoxicity assay was evaluated by the MTS (Promega, Madison, WI, USA) assay. The cytotoxicity assay was evaluated by the 3-(4,5-dimethylthiazol-2-yl)-5-(3-carboxymethoxyphenyl)-2-(4-sulfophenyl)-2H-tetrazolium, inner salt (MTS) (Promega, Madison, WI, USA) assay. Cells were seeded into each well of a 96-well cell culture plate. After 12 h of incubation at 37 °C, the test compound (100 μM) was added. After incubation for 48 h at 37 °C, the cells were subjected to the MTS assay. Compounds with a growth inhibition rate of 50% were further evaluated at concentrations of compound AH106 is re-screened at concentrations of 100, 50, 25, 12.5, 6.25 μM, and the remaining compounds are re-screened at concentrations of 200, 100, 50, 25, 12.5 μM, in triplicate with cisplatin and paclitaxel (Sigma, St. Louis, MO, USA) as positive controls.

The IC_50_ value of each compound was calculated with Reed and Muench's method,^25^ and the results are shown in Table 2.

### X-ray analyses

X-ray crystal data were collected on a Bruker SMART diffractometer equipped with graphite monochromatic Mo *K*_α_ radiation (*λ* = 0.71073 Å) or Cu *K*_α_ radiation (*λ* = 1.34138 Å). The structure was solved using full-matrix least squares on *F*^2^ using the SHELXTL program. All non-H atoms were refined with anisotropic thermal parameters. All hydrogen atoms were located theoretically and refined with riding model position parameters and fixed isotropic thermal parameters. The crystallographic parameters are listed in Table 1.

**Table 1:** Crystal data and refinement parameters for compounds (I)–(VII)

Complex	I	II	III	IV	V	VI	VII
Empirical formula	NiC_15_H_43_N_5_O_111_	Co_3_C_36_H_98_N_6_O_17_	CuC_14_H_32_N_2_O_6_	Cu_2_C_32_H_41_Cl_2_N_4_O_13_	Zn_2_C_24_H_32_Cl_3_N_3_O_3_	Co_3_C_48_H_66_Cl_6_N_6_O_6_	ZnC_18_H_45_Cl_2_N_3_O_3_
Formula mass	528.25	1071.99	387.95	887.67	647.61	1212.55	487.84
Temp (K)	293(2)	293(2)	200(2)	100.0(1)	200(2)	293(2)	293(2)
Wavelength (Å)	0.71073	0.71073	0.71073	1.34138	0.71073	0.71073	0.71073
Crystal system	Orthorhombic	Monoclinic	Monoclinic	Orthorhombic	Monoclinic	Orthorhombic	Orthorhombic
Space group	*P*2_1_2_1_2_1_	*C*2	*P*2_1_	*P*2_1_2_1_2_1_	*P*2_1_	*P*2_1_2_1_2_1_	*P*2_1_2_1_2_1_
*a* (Å)	10.7412(5)	24.5160(11)	4.9600(2)	9.1340(8)	10.264(3)	14.3688(11)	11.3519(12)
*b* (Å)	12.9279(7)	13.4214(6)	22.0851(9)	15.3961(13)	9.025(3)	16.6075(14)	14.6691(13)
*c* (Å)	19.3249(12)	17.9973(6)	8.5898(4)	28.660(3)	15.089(6)	24.609(2)	16.6580(16)
*β* (°)		109.287(1)	98.14(10)		91.457(11)		
Volume (Å^3)^	2683.5(3)	5589.5(4)	931.45(7)	4030.4(6)	1397.3(8)	5872.4(8)	2773.9(5)
*Z*	4	4	2	4	2	4	4
*D*_calcd_ (g cm^−3^)	1.308	1.274	1.383	1.463	1.539	1.372	1.168
*μ* (mm^−1^)	0.779	0.944	1.201	6.872	2.033	1.158	1.097
*F* (000)	1136	2308	414	1828	664	2508	1048
*θ* range (°)	2.61–26.00	2.28–25.50	2.40–26.00	2.83–48.80	1.99–25.50	1.64–25.50	2.27–25.50
Total reflec.	13 299	41 240	9465	115 086	12 859	28 027	23 297
Unique reflections	5246	10 402	3608	5905	5151	10 885	5166
*R*_1_, *wR*_2_ [*I* > 2*σ*(I)]	0.0431, 0.0879	0.054, 0.1307	0.0232, 0.0498	0.0909, 0.2565	0.0721, 0.1713	0.0961, 0.2610	0.0514, 0.1076
*R*_1_, *wR*_2_ [all data]	0.0648, 0.0995	0.0845, 0.1515	0.0255, 0.0512	0.1088, 0.2746	0.1161, 0.1974	0.1363, 0.3040	0.0935, 0.1299
Residuals (e.Å^3^)	0.346,−0.283	0.693,−0.473	0.212,−0.224	0.957,−0.497	1.087,−0.738	1.032,−1.218	0.304,−0.397

**Table 2:** Cytotoxicity of complexes (I)–(VII) against the human tumour cell line A549

Complex	IC_50_^a^ (μM)
I	>200
II	>200
III	31.58 ± 2.80
IV	77.53 ± 1.99
V	17.71 ± 0.45
VI	97.74 ± 5.30
VII	67.61 ± 3.50
*cis*	24.37 ± 0.13

a Cytotoxicity as IC_50_ values for each cell line, the concentration of complex that caused 50% reduction relative to untreated A549 cells determined by the SRB assay. Cisplatin was used as an experimental control.

### Synthesis of complexes (I)–(VII)

#### General experimental details

All reactions were performed in flame-dried glassware under normal atmospheric pressure. Reagents were obtained from commercial sources. Nuclear magnetic resonance (NMR) spectra were acquired on a 500 MHz Bruker Advance III spectrometer. Infrared spectra were recorded on a Mattson Galaxy Series FTIR 3000 spectrometer; peaks were reported in cm–1. Elemental analysis was performed on a VARIO ELIII elemental analyser. The crystal structure was determined by a Gemini S Ultra diffractometer. 1H and 13C NMR chemical shifts were reported in ppm and referenced to CDCl3, 7.26 ppm; for DMSO-daa 6, 2.50 ppm. The following abbreviations were used: s = singlet, d = doublet, t = triplet, q = quartet, m = multiplet. Melting points were measured by Yanaco Micro Melting Point System MP-J3 and SANSYO Melting Point Apparatus SMP-500 and were not corrected.

#### General procedure for the synthesis of complexes (I)–(VII)

The ligand and metal salts (molar ratio of 3 : 1) were heated and refluxed for 48 h, filtration was conducted immediately after the reaction, and the filtrate was kept for slow volatilization. The copper-, cobalt-, nickel- and zinc-containing complexes were successfully synthesized by reacting (*R*)-2-amino-3-methyl butane-1-ol, d-phenyl glycenol and d-leucinol as ligands with Cu(OAc)_2_·H_2_O, Co(OAc)_2_·4H_2_O, Cu(ClO_4_)_2_·6H_2_O, CoCl_2_·6H_2_O and ZnCl_2,_ respectively, and the cultivated crystal was analysed and characterized by X-ray diffraction, IR, ^1^HNMR, ^13^CNMR, UV and E.A. The first step is to find the right ligands, and then, the ligands and the corresponding metal salts reaction was performed on the filter residue or during filtrate processing at the end of the reaction to find a suitable solvent for crystal precipitation. This step is the most critical and requires available solvent tetrahydrofuran, anhydrous methanol, ethanol, and chloroform. Crystals can be frozen in a refrigerator if these substances do not precipitate at room temperature.

#### Synthesis of (*R*)-2-amino-3-methylbutan-1-ol nickel complex (I)

d-Valinol (0.2571 g, 24.9 mmol) and anhydrous methanol (50 mL) were dispersed in a 100 mL round flask, and then Ni(NO_3_)_2_·6H_2_O (0.2415 g, 8.30 mmol) was added to the above solution. The mixture was refluxed for 18 h at 95 °C to 100 °C. After hot filtration, the solution evaporated slowly in the air, and blue crystals were obtained, which were suitable for X-ray single-crystal analysis. The yield was 85%, m. p.160–165 °C. IR (KBr, *ν*, cm^−1^): 3396 (–OH), 3330 (–NH_2_), 2972, (–CH_2_), 1599 (–C=C), 1387 (–C–C), 1339 (–CH_3_), 1162 (–C–O), 1129, 1087, 764, 691, 668 (–Ni–O), 614 (–Ni–N). For [NiC_15_H_43_ N_5_O_11_] anal. calcd., %: C, 34.07; H, 8.14; N, 13.25. Found, %: C, 34.46; H, 7.711; N, 13.32.

#### Synthesis of (*R*)-2-amino-3-methylbutan-1-ol cobalt complex (II)

Complex (II) were synthesized according to general procedure using D-valinol (5.043 g, 48.8 mmol) ligands and Co(OAc) _2_·4H_2_O (4.0517 g, 16.2 mmol) metal salt. The product obtained was dissolved in ethanol and a small amount of DMF for re-crystallization. After 2 days natural evaporation brown-red crystals appeared, which were suitable for X-ray single-crystal analysis. The yield was 86.5%, m. p. 260–262 °C. IR (KBr, *ν*, cm^−1^): 3325 (–OH), 3350 (–NH_2_), 2962 (CH_2_), 1667, 1551 (–C=C), 1403 (–C–C), 1257(–CH_3_), 1188(–C–O), 1057, 1013, 548 (–Co–O), 650 (–Co–N). For [Co_3_C_36_H_98_N_6_O_6_] anal. calcd., %: C, 40.29; H, 9.14; N, 7.83 Found, %: C, 40.44; H, 8.89; N, 8.5%.

#### Synthesis of (*R*)-2-amino-3-methylbutan-1-ol copper complex (III)

For the synthesis of complex (III) followed by general procedure using d-valinol (2.130 g, 206.4 mmol), Cu(OAc)_2_·H_2_O (1.374 g, 6.88 mmol) metal salt. After 3 days natural evaporation the oily product obtained that was further dissolved in diethyl ether and ethyl acetate to re-crystallize the crystal. After natural evaporation blue crystals appeared at the bottom of the vessel, which were suitable for X-ray single-crystal analysis. The yield was 72%, m. p. 205–208 °C. IR (KBr; *ν*, cm^−1^): 3340 (–OH), 3340 (–NH_2_), 2963 (–CH_2_), 1469, 1404 (–C–C), 1339, 1552 (–C=C), 1228 (–CH_3_), 1144 (–C–O), 886, 659 (–Cu–O), 618 (Cu–N). For [CuC_14_H_32_N_2_O_6_] anal. calcd %: C, 43.30; H, 8.24; N, 7.21%. Found, %: C, 43.24; H, 8.682; N, 7.05.

#### Synthesis of (*S*)-2-amino-2-phenylethan-1-ol copper chlorate complex (IV)

Complex IV were synthesize according to general procedure using d-phenyl glycenol (3.2421 g, 23.63 mmol) and Cu(ClO_4_)_2_·6H_2_O (2.9091 g, 7.877 mmol) as a metal salt. After 3 days natural evaporation blue crystals appeared at the wall of the vessel, which was suitable for X-ray single-crystal analysis. The yield was 83.7%, m. p.180–185 °C. IR (KBr; *ν*, cm^−1^):3340 (–OH), 3320 (–NH_2_), 2933 (–CH_2_), 1600 (–C=C), 1496 (–C–C), 1453, 1270 (–CH_3_), 1149 (–C–O), 763, 700, 652 (–Cu–O), 618 (Cu–N). anal. calcd., %: C, 43.25; H, 4.84; N, 6.30%. Found, %: C, 42.85; H, 4.86; N, 6.16.

#### Synthesis of (*S*)-2-amino-2-phenylethan-1-ol zinc complex (V)

Using general procedure the complex (V) were synthesized and characterized using d-phenyl glycenol (0.7781 g, 12.9 mmol) ligand and ZnCl_2_ (0.5888 g 4.32 mmol) as a metal salt in chlorobenzene (40 mL) solvent. The reaction mixture was rotary evaporated, and the residue was dissolved in ethanol and dichloromethane (4; 1), filtered and kept for natural evaporation. After 4 days, white crystals appeared, which were suitable for X-ray single-crystal analysis. The yield was 78.7%, m. p.180–185 °C. IR (KBr, *ν*, cm^−1^): 3360 (–OH), 3340 (–NH_2_), 2941 (–CH_2_), 1600, 1494 (–C–C), 1455 (–C=C), 1397 (–C=0), 1278 (–CH_3_), 1172 (–C–O), 757, 696, 652 (–Zn–O), 588 (–Zn–N). ^1^H NMR (600 MHz, DMSO) *δ* 7.74–7.85 (m, 1H), 7.19–7.30 (m, 4H), 3.98 (s, 1H), 3.68(s, 2H), 3.49 (s, 3H), ^13^C NMR (151 MHz, DMSO and CDCl_3_) *δ* 141.1128.2, 127.3, 127.1, 66.1, 57.2. For [Zn_2_C_24_H_32_Cl_3_N_3_O_3_] anal. calcd., 44.30%; H, 5.56; N, 5.84 Found, %: C, 46.30; H, 5.08; N, 6.38.

#### Synthesis of (*S*)-2-amino-2-phenylethan-1-ol cobalt chloride complex (VI)

Using general method complex (VI) were synthesized and characterized using ligand d-phenyl glycenol (1.082 g, 7.88 mmol) and anhydrous ethanol (40 mL) and CoCl_2_·6H_2_O (0.6253 g, 2.32 mmol) as metal salt. After 3 days natural evaporation, brown-red crystals appeared at the bottom of the beaker, which was suitable for X-ray single-crystal analysis. The yield was 85.9%, m. p. 190–195 °C. IR (KBr; *ν*, cm^−1^): 3370 (–OH), 3355 (–NH_2_), 2860 (–CH_2_), 1615 (–C=C), 1533 (–C=O), 1495 (–C–C), 1454, 1376(–CH_3_), 1194, 1156 (–C–O), 696, 649 (–Co–O), 577 (–Co–N). For [Co_3_C_48_H_66_Cl_6_N_6_O_6_] anal. calcd., %: C, 48.61; H, 5.75, N, 6.52. Found, %: C, 48.87; H, 6.126; N, 7.07.

#### Synthesis of (*R*)-2-amino-4-methylpentan-1-ol zinc chloride complex (VII)

Using general procedure complex (VII) were synthesized consuming d-leucinol (0.9008 g, 7.62 mmol) and ZnCl_2_ (0.349 g, 2.52 mmol) metal salt. After 4 days natural evaporation white crystals appeared at the bottom of the vessel, which were suitable for X-ray single-crystal analysis. The yield was 89%, m. p.70–72 °C. IR (KBr; *ν*, cm^−1^): 3141(–OH), 3317 (–NH_2_), 2955 (–CH_2_) 1716, 1588, 1468 (–C–C), 1388 (–C=C), 1274 (–C=O), 1157 (–CH_3_), 1130 (–C–O), 1022, 688, 648, 592 (–Zn–O), 568 (–Zn–N). ^1^H NMR (600 MHz, DMSO and CDCl_3_) δ 3.78(s, 1H), 3.62 (d, *J* = 8.0 Hz, 1H), 3.29–3.32 (m, 1H), 1.73–1.75 (m, 1H), 1.26–1.36(m, 2H), 0.91, 0.93 (dd, *J* = 6.6, 6.5 Hz, 6H); ^13^C NMR (150 MHz, DMSO and CDCl_3_) 64.6, 61.4, 51.0, 41.8, 29.5, 24.4, 22.9, 22.7. For [ZnC_18_H_45_Cl_2_N_3_O_3_] anal. calcd., %: C, 44.30; H, 9.30; N, 8.61%. Found, %: C, 44.45; H, 8.94; N, 8.30.

### X-ray structure

X-ray diffraction data for complex (I)–(VII) were collected at room temperature using graphite-monochromatic Mo k_α_ radiation (*λ* = 0.71073 Å) on an Oxford Diffraction GeminiS diffractometer. Structure solution and refinement for complex I–VII were carried out with the programs SHELXT^26^ and SHELXL-2018/3,^27^ respectively. MERCURY^28^ was employed for molecular graphics and OLEX2.^29^ Non-hydrogen atoms in (I)–(VII) were refined anisotropically while hydrogen atoms were treated by constrained isotropic refinement. Crystal data and refinement parameters for compounds (I)–(VII) are summarized in Table 1. The selected bond lengths and bond angles are listed in Table S1.† Hydrogen bonds of the complex (I)–(VII) is given in Table S2.†

### Description of the crystal structures for complexes (I)–(VII)

**Fig. 1:**
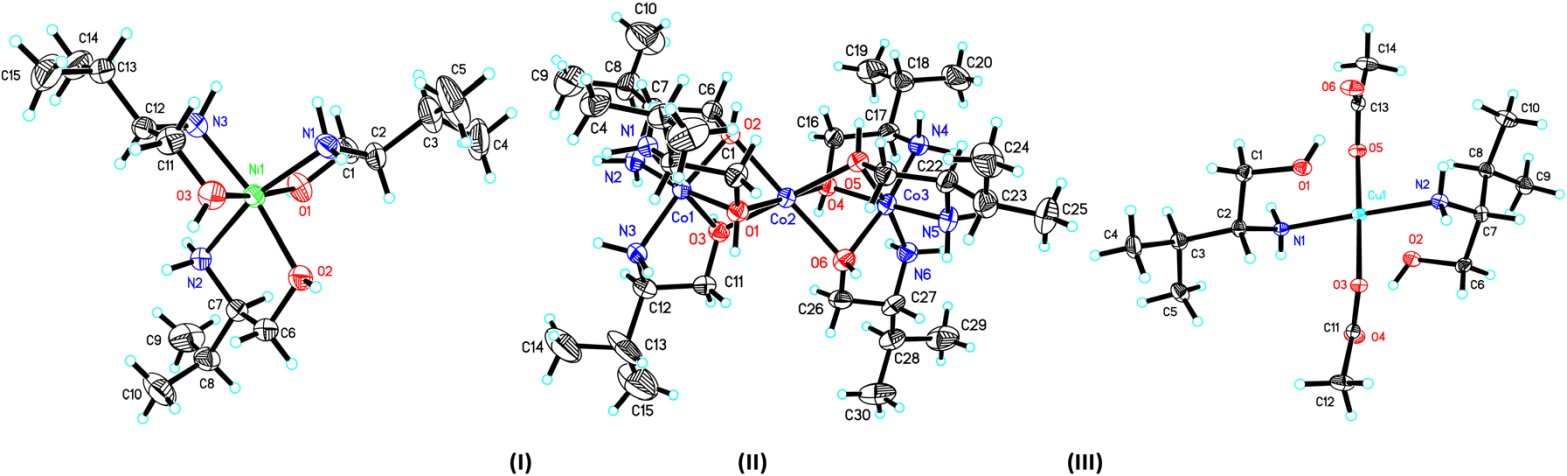
The ORTEP molecular structures of complexes (I) to (III) shown as 30% thermal ellipsoid probabilities.

**Fig. 2:**
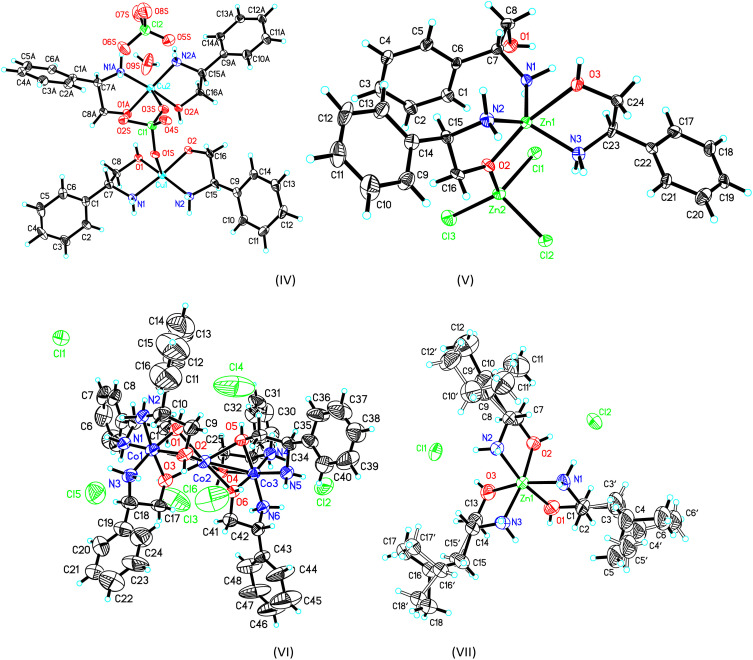
The ORTEP molecular structures of complexes (IV) to (VII) shown as 30% thermal ellipsoid probabilities.

The crystal structure and stereogram of complexes (I)–(III) is shown in Fig. 1 and (IV)–(VII) in Fig. 2. All the complexes from (I) to (VII) are crystallized in the chiral space groups under a certain experimental conditions, *i.e.*, *P*2_1_2_1_2_1_ for (I)/(II)/(VI)/(VII), *P*2_1_ for (III)/(V), and *C*2 for (II), respectively (Table 1). For mono-nuclear metal complex (I), (III), (IV) and (VII), there are one (for (I), (III) and (VII)) or two (for IV)) metal ions, two (for (III)), three (for (I), (VII)) or four (for (IV)) ligands, two counter-anions and two water molecules (for (I)) in their asymmetric units. For compound (V), it is a di-nuclear metal coordination complexes, in whose asymmetric units there is two zinc ions, each three d-phenyl glycenol and three chloride anions. For compound (II) and (VI), they are both in a fashion of tri-nuclear cobalt coordination modes except for the main ligands being different. In their asymmetric units, there are three cobalt cations, six main ligands, three (for (II)) or six (for (VI)) counter anions and several solvent molecules.

In the crystal of complex (I), the central nickel ion adopts the octahedron coordination sphere by a N_3_O_3_ donor set from three separated d-valinol molecules. The bond lengths (*d*_Ni–N_ = 2.071(4) Å/2.085(4) Å, *d*_Ni–O_ = 2.076(3) Å −2.130(3) Å) and angles (79.95(4)° − 98.42(16)° for the *cis* angles and 164.77(16)° − 171.41(13)° for the *trans* angles around nickel centre) are comparable to some analogs.^30,31^ In its crystal packing, the molecules are linked into a three-dimensional hydrogen-bonded network by a combination of N–H⋯O and O–H⋯O interactions (Table S2 and Fig. S1†).

The crystal of complex (II), these three metal centres are linked three μ_2_-O bridges between each two cobalt ions with the Co_1_⋯Co_2_ and Co_2_⋯Co_3_ distances of 2.644(2) Å and 2.637(2) Å, respectively. For the two lateral cobalt atoms, they both are in the octahedron coordination environments surrounded by each three nitrogen atoms and three oxygen atoms from three d-valinol ligands. But the middle Co_3_ is best described as in an inclined trigonal prism coordination by six oxygen atoms. Similar to those in (I), the bond lengths and angles around three cobalt centres are also comparable to its analogs.^32^ The component ions in (II) are joined together, forming the final three-dimensional network by the extensive hydrogen bonds (Table S2 and Fig. S1†).

In the crystal of complex (III), the central metal copper atom gives a square-planar coordination by each two d-valinol nitrogen atoms and two acetate oxygen atoms when the two valinol oxygen atoms are not considered due to their far distant away from the Cu_1_ atom [2.493(3) Å/2.502(3) Å]. The four bond lengths are *d*_Cu1–N_1__ = 1.997(3) Å, *d*_Cu1–N_2__ = 2.000(3) Å, *d*_Cu1–O_1__ = 2.003(3) Å, and *d*_Cu1–O_2__ = 2.020(2) Å. And the four *cis* and two *trans* angles around copper centre are of 89.08(14)°/88.57(11)° and 177.33(16)°/178.11(12)°, respectively. In the crystal packing, the molecules are linked into a one-dimensional hydrogen-bonded chain along the [100] axis (Table S2 and Fig. S1†).

For the crystal of complex (IV), because the perchlorate O_1_S, O_3_S and water O_9_S atoms are distant more than 2.60(1) Å away from the central metal atoms, the coordination of the both the copper atoms can be described as square-planar configurations. The Cu–N (or O) bond lengths are in a range of 1.935(8) Å to 2.007(10)Å around Cu_1_, and 1.911(11)Å to 1.986(14) Å around Cu_2_. The *cis* and *trans* angles are of 85.40(1)° to 96.35(1)° and 170.55(1)° to 173.62(4)°, respectively, around Cu_1_ atom. Those *cis* and *trans* angles are of 84.82(4)° to 98.36(4)° and 173.50(4)° to 175.05(4)°,respectively, around Cu_2_ atom which are comparable with some analogs.^33^ It should be mentioned that one of the coordinated d-phenyl glycenol is disprotonated when coordinating the copper atom and its Cu–O bond lengths are slightly shorter than the un-disprotonated Cu–O bond. These two discrete coordination cations are joined together into a dimmer by a complementary O–H⋯O hydrogen bonds. Additionally, the molecules are linked into three-dimensional network by a combination of N–H⋯O hydrogen bonds in the crystal packing (Table S2 and Fig. S1†).

The crystal of complex (V) was composed of a di-nuclear zinc complex in which these two metals are joined together by a μ_2_-O bridge from a d-phenyl glycenol molecule and the Zn_1_⋯Zn_2_ distance is 3.444(4) Å. For Zn_1_ and Zn_2_, they adopt the bi-pyramidal and tetrahedral coordination sphere, respectively. As for the bi-pyramidal polyhedron around Zn_1_, two glycenol oxygen O_2_ and O_3_ atoms are positioned at the apical sites and the three amine nitrogen atoms locate at the basal plane. As for Zn_2_ atoms, its tetrahedral coordination is composed of three chloride anions and one glycenol oxygen atom. The coordination bond lengths are ranging from 2.028(11) Å to 2.085(12) Å around Zn_1_ and from 1.918(11) Å to 2.345(4) Å around Zn_2_. The *cis* bond angles are varying from 73.93(1)° to 127.32(4)° and the *trans* angle is 165.45(4)° around Zn_1_ atom. For Zn_2_ atom, the angles are ranging from 99.61(4)° to 119.37(4)°. In the crystal packing, the molecules are linked into a two-dimensional layer structure by the N–H⋯O and O–H⋯O hydrogen bonds running parallel to the (001) plane (Table S2 and Fig. S1†).

In the crystal of complex (VI), the tri-nuclear coordination core is similar to that in complex (II) except for the ligand is different. The Co_1_⋯Co_2_ and Co_2_⋯Co_3_ distances are both 2.650(4) Å which is slightly longer those in crystal of (II). No more discussions are given due to its structural similarity with crystal (II). The molecules are also linked into the final three-dimensional network by the extensive N–H⋯Cl and O–H⋯Cl hydrogen bonds.

For the crystal of (VII), the mono-nuclear central zinc metal atom adopts the octahedral coordination configuration furnished by a N_3_O_3_ donor set. The bond lengths and angles are in a range from 2.094(6) Å to 2.203(5) Å and from 78.70(4) to 100.14(4) which are similar to its analogs.^34–36^ the component ions are linked into a three-dimensional network by a combination of O–H⋯Cl and N–H⋯C_l_ hydrogen bonds (Table S2 and Fig. S1†).

## Results

### NMR spectroscopy for complexes (V) and (VII)

**Fig. 3:**
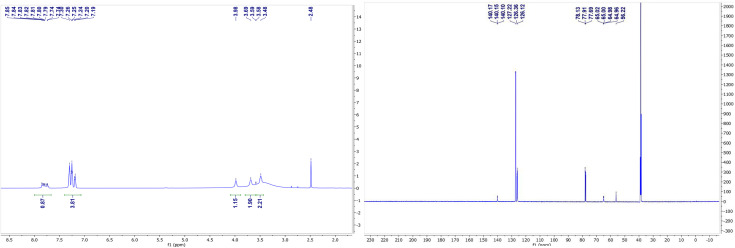
^1^H NMR (left) and ^13^C NMR (right) spectrum of complex V.

**Fig. 4:**
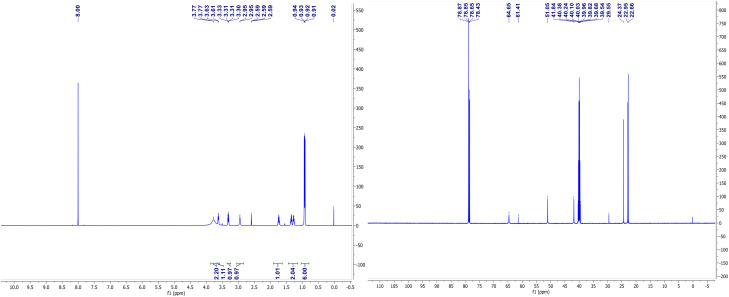
^1^HNMR and ^13^CNMR spectrums of complex (VII).

The NMR spectra of complexes (V) and (VII) are shown in Fig. 3 and 4. For the zinc complexes, NMR for complexes (V) and (VII) were also tested further, and from Fig. 3 and 4, we can see that *δ* 7.76–7.85 and 7.19–7.30 ppm are the characteristic absorption peaks of five protons in the benzene ring. Additionally, δ 3.98, 3.69 and 3.49 ppm are absorption peaks of the CH, CH_2_ and NH_2_ group H protons, respectively. In the ^13^CNMR, δ 140.1, 127.2, 126.3, 126.1, 65.0, and 56.2 ppm are also characteristic absorption peaks, including phenyl carbon protons, tertiary carbon and methylene carbon protons. Similar to complex (VII), in the ^1^HNMR, *δ* 3.78, 3.62, 3.30–3.33, and 2.94 each represent the characteristic absorption peaks of the H protons of the CH, CH_2_ and NH_2_ and isobutyl groups (*δ* 1.74, 1.26–1.36, 0.91 and 0.94 ppm). All the NMR results, whether in ^1^HNMR or ^13^CNMR, confirmed that the exact structures of complexes (V) and (VII) are consistent with the analytical results of the crystal structures.

### IR spectroscopy of complexes (I)–(VII)

**Fig. 5:**
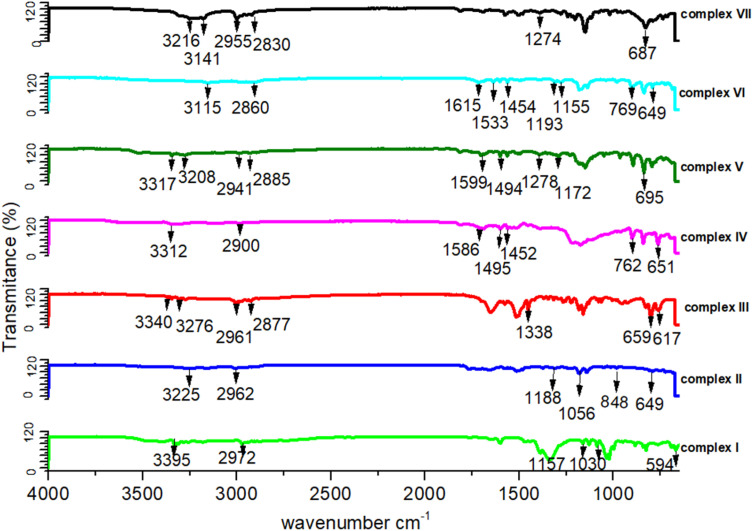
IR spectra of all the complexes (I) to (VII) in range of 4000–500 cm^−1^ region.

The IR analysis shows a number of peaks that are in all IR spectra (shown in Fig. 5). The IR vibrations of the OH group are usually found to be most sensitive to the environment. As such, they show distinct shifts in the spectra of the hydrogen-bonded species. Generally, the optimum absorption region of non-hydrogen bonded or a free hydroxyl group is in a range of 3550–3700 cm^−1^. In the case of their presence in a molecule, intra and intermolecular hydrogen bonding reduces the O–H stretching band to the region of 3000–3550 cm^−1^, signifying the O–H⋯O interactions in the crystal packing. In all our studied complexes, an obvious red shift phenomenon of the hydroxyl groups have been also observed for the infrared absorption vibration peak at 3115–3395 cm^−1^, which once more demonstrates the weakening of the O–H bond energy due to the delocalization of electrons through hydrogen bonds. The C–C vibrations (1200 cm^−1^–1400 cm^−1^) have been shifted toward some regions of very large wavelengths upon complexation, showing that the electron density in C–C bond is increased by transition metal complexation by the stated ligands. Some sharp peaks at 3310–3350 cm^−1^ representing the existence of NH_2_ groups. For these functional groups such as all the C–H bonds, their infrared absorption peaks lie between 3000 and 2000 cm^−1^, C=O and C=C stretching vibrations are at almost 1720 and 1600 cm^−1^, and C–O vibrations are at 1280 and 1090 cm^−1^, respectively.^37,38^ In more details, there is the 3100 to 3000 cm^−1^ peak of the aromatic C–H stretching vibration, the stronger absorption at 1600 cm^−1^, which can be referred to as aromatic C=C bonds, and aliphatic C–H stretching vibrations between 3000 and 2800 cm^−1^. The presence of aromatic structures has been supported by the existence of the spectral region between 1500 and 1630 cm^−1^. The absorbance bands at 1300 cm^−1^ to 1400 cm^−1^ can be assigned to bending vibrations of the CH_3_ group. These peaks at 1465 cm^−1^ and 1150 cm^−1^ are dominated by the C–O stretching vibrations.^39–41^ There are stretching vibration peaks at 600–650 cm^−1^ and 500–600 cm^−1^ for metal-nitrogen and stretching vibration peaks at 594 cm^−1^, 659 cm^−1^, 617 cm^−1^, 649 cm^−1^, 848 cm^−1^ and 695 cm^−1^ for Ni–N, Cu–N, Cu–O, Co–N, Co–O and Zn–N, respectively, that further signifies the interactions between metal and ligands. A peak appeared at 650–670 cm^−1^ for the C–Cl band and 1500–1600 cm^−1^ for ClO_4_ (IR figures are shown in Fig. S2†).

### UV-vis spectroscopy for complexes (I)–(VII)

**Fig. 6:**
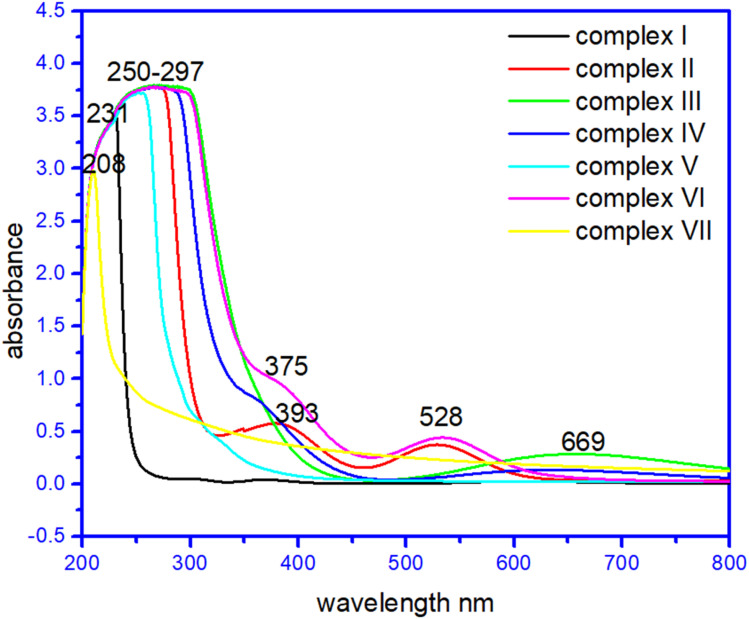
UV visible spectra of complexes (I) to (VII).

In their methanol solutions, the absorption spectra of the seven complexes and raw materials have been reported.^42–45^ Transition metal complexes are often coloured owing to the presence of multiple electronic states associated with incompletely filled d orbitals. Evidence of coordination of the metal ion with the corresponding ligands was provided by the d–d transitions of the metal ion observed in the complexes, which were absent in the ligands, the strong band in the ultraviolet region was ascribed to the n → π* transition of the ligands. Interestingly, the complex (I) at 230–234 nm, complex (II) 260–264 nm, 375–379 nm, and 525–527 nm and complex (VII) has broad peaks at 210–213 and 280–282 nm due to σ → σ*,and n → σ* C–O/C–N transition. Whereas complex (III) has a broad band at 278–281 nm due to σ → σ*, and n → σ* C–O/C–N transition while 640–669 nm due to C=O n → π* transition. Surprisingly complex (IV), complex (V) and complex (VI) has peaks at 268–271 nm and 360–363 nm, and a broad peak at 250–253 nm. Complex (VI) has a large peak at 255–257 nm, 386–388 nm, and 525–527 nm due to σ → σ*,and n → σ * C–O/C–N transition and due to n → π* C=C transition, respectively.^27–29^ The UV spectra of the complexes are given in Fig. 6. The UV spectra of the complexes (I)–(VII) are given in Fig. S3†.

In order to ascertain the colour origin of the complexes, it can be deduced from the crystal-field-theory view. As for a first-transition metal atom, its five 3d-orbitals can be splitted into two sets in a certain formed crystal field due to the interactions of the various outside ligands. From Fig. 6, the apparent d–d transitions of compounds (II), (III) and (VI) can be easily observed with their respective maximum absorptions at 528 nm, 528 nm and 669 nm. As is well known that by a combined effect from six ligands in octahedral coordination compounds (II) and (VI), the 3d orbitals for the first-transition metal will be energetically splitting into two groups, three irreducible lower energetic *t*_2g_ (d_*xy*_, d_*yz*_ and d_*xz*_) and two higher energetically e_2g_ (d_*x*_2_−*y*_2__, d_*z*_2__) ones. Then, the electron-transition in the visible light region should occur between these two groups of obitals. However, the copper atom in compound (III) was four-coordinated which results in its 3d orbitals splitting into four sets in terms of energetic sequence d_*yz*_/d_*xz*_, d_*z*_2__, d_*xy*_ and d_*x*_2_−*y*_2__. These d–d transitions absorb light in visible region, it can make the compounds present its complementary colours such as the red-brown for compound (II) and (VI) and blue for compound (III).

### Cytotoxicity assay

The anticancer activity of amino alcohol complexes (I)–(VII) showed cytotoxic effects against the human tumour cell line A549; among them, complex (V) showed the best activity with an IC_50_ value of 17.8. The IC_50_ values of the seven compounds along with cisplatin were tested and calculated *via* Reed and Muench's method.^25^ In order testify the stabilities of these compounds, we have tested all their UV-vis absorption spectra in a certain solution medium (Fig. S4†) over time process. The results indicate that they didn't undergo apparent change after an overnight standing in air condition, which also clearly proved that the compounds were stable in their cytotoxicity research.

From a structural point, we found that the type of metal coordination, the small guest ligands around a metal center and the coordination unsaturation degree are all related to their activities. For example, the activity of zinc compound V is in generally greater than that of copper compound III, which is in turn apparently greater than that of cobalt and nickel compounds II, VI and I. For compound V, its anticancer activity is better than that of compound VII which should be due to the presence of a metal zinc centre coordinated by three chlorine atoms and one oxygen atom. The three coordinated chloride ions may be more easily hydrolyzed during the process of its anticancer activity. For the two copper compounds III and VI, the copper ion in III is more easily exposed to a certain substrate groups due to its planar four-coordinated geometry. Perhaps for this reason, compound III also showed better anticancer activity with the IC_50_ value of 31.58 ± 2.80 μM, which is kind of like the mechanism of the four-coordinated platinum atom in cisplatin compound. Further relationship between the coordination environment of a metal atom and its activities are ongoing in our lab.

## Conclusions

A series of Zn(ii), Cu(ii), Co(ii), and Ni(ii) novel complexes containing chiral amino alcohols were synthesized through one-pot method, and characterized spectroscopically using NMR, FTIR, UV-visible, and EA as well as single-crystal X-ray diffraction techniques to confirm the structure of already synthesized chiral amino alcohol complexes. The complexes were then evaluated against anti-lungs cancer cell. They also exhibited cytotoxic activities against A549 cell lines. Compared to the others, complex (V) had an IC_50_ of 17.8 against A549 lung cancer cells. The observations demonstrated that the anticancer activity of these complexes is dependent on the type of metal ion, cell line, and geometries of the corresponding molecule. The obtained anticancer activities results are mark able and comparable to the available anti-cancer drugs in market. We expect that these complexes could be a remarkable drug in future and replace cis-pt as well as use for other medicinal application. The intriguing results may help in the design and development of new medicinal drug-like compounds. The catalytic activities of complexes (I)–(VII) for different organic reactions are currently ongoing.

## Author contributions

M. Luo designed the research, analysed the data and revised the article; H. Yin and J. C. Zhang helped with NMR and elemental testing. Q. Umar wrote the draft, performed the research work and contributed equally with Y. H. Huang to this research work. Y. H. Huang and A. Nazeer also performed the research work. X.-G. Meng revised the draft and analysed the data; all authors read and approved the final manuscript.

## Consent for publication

All authors consent to the publication.

## Conflicts of interest

The authors declare that they have no competing interests.

## Supplementary Material

RA-012-D2RA05576G-s001

RA-012-D2RA05576G-s002
