# Supplementary material for: Synthesis, characterization and anticancer activities of Zn2+, Cu2+, Co2+ and Ni2+ complexes involving chiral amino alcohols
Source: RSC Adv. 2022 Nov 9;12(49):32119–28. doi: 10.1039/d2ra05576g (PMC9644435; doi:10.1039/d2ra05576g)
Supplement: RA-012-D2RA05576G-s001 [file RA-012-D2RA05576G-s001.pdf]

Electronic Supplementary Material (ESI) for RSC Advances.
This journal is © The Royal Society of Chemistry 2023

Supporting Information

Synthesis, characterizations and Anticancer Activities of Zn^{2+}, Cu^{2+}, Co^{2+} and Ni^{2+} Complexes Involving Chiral Amino Alcohols

Q. Umar[a], Y. H. Huang[a], A. Nazeer[a], H. Yin[b], J. C. Zhang[a], M. Luo[a*], X. G. Meng[c*]

[a] Department of Chemistry and Chemical Engineering, Hefei University of Technology, Hefei, 23000, P.R. China

[b] Department of Chemistry and Chemical Engineering, Hefei University of Technology, Hefei, 23000, P.R. China

[c] College of Chemistry, Central China Normal University, Wuhan, 430079, P.R. China

Corresponding Author: M. Luo, e-mail: luomei@pku.edu.cn; mengxianggao@ccnu.edu.cn

Q. Umar and Y. H. Huang contributed equally to this work

Contents of the supporting information:

Table S1 The selected Bond lengths (Å) and angles (°) for complexes (I) - (VII).

Table S2 Hydrogen Bond Lengths (Å) and Bond Angles (°) for complexes (I) - (VII).

Figure S1 The crystal packing of complexes (I)-(VII), showing the formation of the three-dimensional network by hydrogen bonding interactions, (a) for (I), (b) for (II), (c) for (III), (d) for (IV), (e) for (V), (f) for (VI) and (g) for (VII), respectively.

Figure S2 IR spectra of all the complex (I) - (VII) in the ranges of 4000-500 cm^{-1}, showing individual infrared absorption spectrum of each compound.

Figure S3 UV spectra of complexes (I) - (VII) in the ranges of 200-800 nm, showing individual UV-Vis absorption spectrum of each compound.

Table S1 Selected bond lengths [Å] and angles [°] for complexes (I) – (VII).

		**Complex (I)**			
**Ni1-N1**	**2.071(4)**	**Ni1-O1**	**2.078(3)**	**Ni1-N2**	**2.085(4)**
Ni1-O3	2.076(3)	Ni1-N3	2.085(4)	Ni1-O2	2.130(3)
N1-Ni1-O3	91.69(16)	O3-Ni1-N3	81.43(14)	O3-Ni1-N2	97.90(14)
N1-Ni1-O1	79.95(15)	O1-Ni1-N3	97.75(15)	O1-Ni1-N2	90.69(14)
O3-Ni1-O1	171.41(13)	N1-Ni1-N2	164.77(16)	N3-Ni1-N2	94.70(15)
N1-Ni1-N3	98.42(16)	N1-Ni1-O2	88.32(15)	O3-Ni1-O2	88.48(14)
O1-Ni1-O2	93.19(15)	N3-Ni1-O2	167.99(14)	N2-Ni1-O2	80.17(14)
		**Complex (II)**			
Co1-N1	1.931(6)	Co1-N2	1.942(6)	Co1-N3	1.943(6)
Co1-Co2	2.644(12)	Co2-O4	2.056(5)	Co2-O3	2.078(5)
Co2-O5	2.088(5)	Co2-O1	2.117(5)	Co2-O6	2.127(6)
Co2-O2	2.127(5)	Co2-Co3	2.63(13)	Co3-O4	1.897(5)
Co3-O6	1.901(6)	Co3-O5	1.908(5)	Co3-N4	1.937(7)
Co3-N5	1.940(7)	Co3-N6	1.948(6)		
O2-Co1-O1	85.8(2)	N3-Co1-Co2	121.0(2)	O3-Co2-Co1	46.11(14)
O2-Co1-O3	86.3(2)	O4-Co2-O1	177.0(2)	O5-Co2-Co1	135.72(15)
O1-Co1-O3	86.7(2)	O3-Co2-O1	77.8(2)	O1-Co2-Co1	45.88(13)
O2-Co1-N1	87.1(2)	O5-Co2-O1	105.0(2)	O4-Co3-N4	85.5(3)
O1-Co1-N1	93.3(2)	O4-Co2-O6	76.3(2)	O6-Co3-N4	171.0(3)
O3-Co1-N1	173.8(2)	O3-Co2-O6	104.5(2)	O5-Co3-N4	92.0(3)
O2-Co1-N2	86.5(2)	O5-Co2-O6	75.4(2)	O4-Co3-N5	173.7(3)
O1-Co1-N2	172.3(3)	O1-Co2-O6	105.3(2)	O6-Co3-N5	93.5(3)
O3-Co1-N2	92.6(2)	O4-Co2-O2	102.9(2)	O5-Co3-N5	87.3(3)
N1-Co1-N2	93.6(3)	O3-Co2-O2	76.95(19)	N4-Co3-N5	94.9(3)
O2-Co1-N3	172.9(3)	O5-Co2-O2	103.1(2)	O4-Co3-N6	91.6(3)
O1-Co1-N3	92.4(3)	O1-Co2-O2	75.64(19)	O6-Co3-N6	87.0(3)
O3-Co1-N3	86.7(2)	O6-Co2-O2	178.4(2)	O5-Co3-N6	172.0(3)
N1-Co1-N3	93.5(3)	O4-Co2-Co3	45.62(15)	N4-Co3-N6	95.5(3)
N2-Co1-N3	95.2(3)	O3-Co2-Co3	132.20(14)	N5-Co3-N6	94.6(3)
O2-Co1-Co2	52.76(15)	O5-Co2-Co3	45.84(14)	O4-Co3-Co2	50.79(16)
O1-Co1-Co2	52.39(15)	O1-Co2-Co3	137.23(14)	O6-Co3-Co2	52.92(16)
O3-Co1-Co2	51.22(15)	O6-Co2-Co3	45.49(15)	O5-Co3-Co2	51.70(16)
N1-Co1-Co2	124.1(18)	O2-Co2-Co3	132.99(15)	N4-Co3-Co2	119.0(2)
N2-Co1-Co2	121.8(2)	O4-Co2-Co1	131.28(15)	N5-Co3-Co2	124.2(2)
N6-Co3-Co2	121.6(2)	O6-Co2-Co1	136.05(16)	O2-Co2-Co1	45.51(14)
		**Complex (III)**			
Cu1-O5	2.000(3)	Cu1-O3	2.020(2)	Cu1-O2	2.502(3)
Cu1-N2	2.003(3)	Cu1-O1	2.493(3)		
N1-Cu1-O5	89.41(10)	N2-Cu1-O3	88.57(11)	N1-Cu1-O2	103.47(11)
N1-Cu1-N2	177.33(16)	N1-Cu1-O1	77.88(11)	O5-Cu1-O2	90.10(9)
O5-Cu1-N2	92.97(14)	O5-Cu1-O1	91.94(9)	N2-Cu1-O2	77.74(11)
N1-Cu1-O3	89.08(14)	N2-Cu1-O1	100.83(11)	O3-Cu1-O2	89.15(9)
O5-Cu1-O3	178.11(12)	O3-Cu1-O1	88.86(9)	O1-Cu1-O2	177.57(7)
		**Complex (IV)**			
Cu1-N1	1.987(11)	Cu1-O1S	2.770(10)	Cu2-O2A	1.967(9)
Cu1-N2	2.007(10)	Cu2-N1A	1.976(12)	Cu2-O3S	2.597(11)

Cu1-O1	1.954(9)	Cu2-N2A	1.986(14)	Cu2-O9S	2.71(3)
Cu1-O2	1.935(8)	Cu2-O1A	1.911(11)		
N1-Cu1-N2	96.3(4)	O2-Cu1-N1	173.6(4)	N1A-Cu2-O9S	96.9(8)
N1-Cu1-O1S	84.4(4)	O2-Cu1-N2	86.0(4)	N2A-Cu2-O3S	85.6(6)
N2-Cu1-O1S	81.1(4)	O2-Cu1-O1	93.3(4)	N2A-Cu2-O9S	82.9(11)
O1-Cu1-N1	85.4(4)	O2-Cu1-O1S	90.1(3)	O1A-Cu2-N1A	84.9(5)
O1-Cu1-N2	170.6(4)	N1A-Cu2-N2A	98.3(6)	O1A-Cu2-N2A	175.1(7)
O1-Cu1-O1S	108.3(3)	N1A-Cu2-O3S	88.4(5)	O1A-Cu2-O2A	91.6(5)
O1A-Cu2-O3S	98.3(4)	O1A-Cu2-O9S	93.0(10)	O2A-Cu2-N1A	173.5(5)
O2A-Cu2-N2A	85.6(5)	O2A-Cu2-O3S	86.8(4)	O2A-Cu2-O9S	88.7(8)
O3S-Cu2-O9S	167.9(8)				

Complex (V)

Zn1-O2	2.028(11)	Zn1-N2	2.085(12)	Zn2-Cl3	2.227(5)
Zn1-N1	2.047(13)	Zn1-O3	2.310(10)	Zn2-Cl1	2.283(4)
Zn1-N3	2.068(13)	Zn2-O2	1.918(11)	Zn2-Cl2	2.345(4)
O2-Zn1-N1	109.5(5)	N1-Zn1-N2	122.0(5)	N3-Zn1-O3	73.9(5)
O2-Zn1-N3	97.2(5)	N3-Zn1-N2	127.3(5)	N2-Zn1-O3	93.1(4)
N1-Zn1-N3	107.6(5)	O2-Zn1-O3	165.5(5)	O2-Zn2-Cl3	114.5(3)
O2-Zn1-N2	83.1(4)	N1-Zn1-O3	84.5(5)	O2-Zn2-Cl1	102.0(3)
Cl3-Zn2-Cl1	119.37(19)	O2-Zn2-Cl2	109.3(4)	Cl3-Zn2-Cl2	110.68(18)
Cl1-Zn2-Cl2	99.61(16)				

Complex (VI)

Co1-O3	1.883(9)	Co1-N1	1.946(12)	Co2-O3	2.095(10)
Co1-O1	1.900(10)	Co1-N3	1.958(12)	Co2-O5	2.111(9)
Co1-O2	1.920(10)	Co1-Co2	2.650(2)	Co2-O2	2.112(9)
Co1-N2	1.937(11)	Co2-O6	2.058(8)	Co2-O1	2.113(9)
Co2-O4	2.125(9)	Co2-Co3	2.649(2)	Co3-O6	1.882(9)
Co3-N6	1.902(11)	Co3-O4	1.905(9)	Co3-O5	1.945(8)
Co3-N5	1.958(10)	Co3-N4	1.988(11)		
O3-Co1-O1	86.0(4)	O3-Co1-Co2	51.7(3)	O2-Co2-O1	75.8(3)
O3-Co1-O2	86.7(4)	O1-Co1-Co2	52.2(3)	O6-Co2-O4	75.9(3)
O1-Co1-O2	85.6(4)	O2-Co1-Co2	52.1(3)	O3-Co2-O4	100.5(3)
O3-Co1-N2	173.8(5)	N2-Co1-Co2	122.6(4)	O5-Co2-O4	77.4(3)
O1-Co1-N2	91.9(5)	N1-Co1-Co2	121.5(3)	O2-Co2-O4	175.6(4)
O2-Co1-N2	87.3(4)	N3-Co1-Co2	121.1(4)	O1-Co2-O4	106.9(3)
O3-Co1-N1	91.5(4)	O6-Co2-O3	108.0(4)	O6-Co2-Co3	45.0(2)
O1-Co1-N1	86.9(4)	O6-Co2-O5	76.3(3)	O3-Co2-Co3	134.6(2)
O2-Co1-N1	172.4(5)	O3-Co2-O5	174.7(3)	O5-Co2-Co3	46.5(2)
N2-Co1-N1	94.3(5)	O6-Co2-O2	101.7(3)	O2-Co2-Co3	134.8(3)
O3-Co1-N3	88.0(4)	O3-Co2-O2	76.7(4)	O1-Co2-Co3	134.5(3)
O1-Co1-N3	173.2(4)	O5-Co2-O2	105.7(4)	O4-Co2-Co3	45.4(2)
O2-Co1-N3	90.8(5)	O6-Co2-O1	175.2(4)	O6-Co2-Co1	135.3(3)
N2-Co1-N3	93.7(5)	O3-Co2-O1	75.6(4)	O3-Co2-Co1	44.9(2)
N1-Co1-N3	96.6(5)	O5-Co2-O1	100.3(4)	O5-Co2-Co1	133.9(2)
O2-Co2-Co1	45.9(3)	O6-Co3-N6	88.6(4)	N6-Co3-O5	173.2(4)
O1-Co2-Co1	45.3(3)	O6-Co3-O4	85.6(3)	O4-Co3-O5	86.9(4)
O4-Co2-Co1	133.9(2)	N6-Co3-O4	92.5(4)	O6-Co3-N5	91.6(5)
Co3-Co2-Co1	179.29(9)	O6-Co3-O5	84.6(4)	N6-Co3-N5	92.9(5)
O4-Co3-N5	173.8(4)	O5-Co3-N5	87.3(4)	O6-Co3-N4	171.6(4)

N6-Co3-N4	93.1(5)	O4-Co3-N4	86.1(4)	O5-Co3-N4	93.6(4)
N5-Co3-N4	96.6(5)	O6-Co3-Co2	50.6(2)	N6-Co3-Co2	122.8(3)
O4-Co3-Co2	52.6(3)	O5-Co3-Co2	52.0(3)	N5-Co3-Co2	121.6(4)
N4-Co3-Co2	122.3(3)				
		**Complex (VII)**			
Zn1-N1	2.094(6)	Zn1-O3	2.191(6)	Zn1-O2	2.194(5)
Zn1-N3	2.101(6)	Zn1-O1	2.203(5)	Zn1-N2	2.105(6)
N1-Zn1-N3	98.6(3)	N2-Zn1-O3	94.8(2)	N1-Zn1-O1	78.7(2)
N1-Zn1-N2	100.1(2)	N1-Zn1-O2	94.5(2)	N3-Zn1-O1	94.2(2)
N3-Zn1-N2	99.3(3)	N3-Zn1-O2	166.9(3)	N2-Zn1-O1	166.5(2)
N1-Zn1-O3	165.1(2)	N2-Zn1-O2	79.4(2)	O3-Zn1-O1	86.8(2)
N3-Zn1-O3	78.8(3)	O3-Zn1-O2	88.2(2)	O2-Zn1-O1	87.2(2)

Table S2 Hydrogen bond lengths (Å) and bond angles (°) for complexes (I) - (VII).

D–H···A	d(D–H)	d(H···A)	d(D···A)	∠DHA
		**Complex (I)**		
O10-H10D...O4	0.85(3)	2.19(9)	2.752(7)	123(9)
O11-H11D...O4	0.85	2.08	2.816(6)	144.7
O10-H10E...O8	0.85	2.31	3.119(6)	159.1
C12-H12...O5$^{\#1}$	0.98	2.57	3.517(7)	163.8
N3-H3B...O6$^{\#2}$	0.97	2.41	3.273(6)	147.6
N3-H3A...O7$^{\#3}$	0.97	2.32	3.258(6)	162.6
N2-H2B...O6$^{\#2}$	0.97	2.18	3.065(6)	151.8
N2-H2A...O5$^{\#1}$	0.97	2.22	3.128(6)	155.0
N1-H1B...O7	0.97	2.11	3.065(6)	168.9
N1-H1A...N5$^{\#3}$	0.97	2.70	3.657(7)	171.0
N1-H1A...O9$^{\#3}$	0.97	2.17	3.104(6)	162.1
O3-H3...O11	0.856(12)	1.80(2)	2.610(5)	158(5)
O2-H2...O8	0.870(13)	1.92(2)	2.767(5)	165(4)
O1-H1...O10$^{\#2}$	0.848(12)	1.766(17)	2.608(5)	172(6)
O1-H1...O10$^{\#2}$	0.848(12)	1.766(17)	2.608(5)	172(6)
O2-H2...O8	0.870(13)	1.92(2)	2.767(5)	165(4)
O3-H3...O11	0.856(12)	1.80(2)	2.610(5)	158(5)
N1-H1A...O9$^{\#3}$	0.97	2.17	3.104(6)	162.1
N1-H1A...N5$^{\#3}$	0.97	2.70	3.657(7)	171.0
N1-H1B...O7	0.97	2.11	3.065(6)	168.9
N2-H2A...O5$^{\#1}$	0.97	2.22	3.128(6)	155.0
N2-H2B...O6$^{\#2}$	0.97	2.18	3.065(6)	151.8
N3-H3A...O7$^{\#3}$	0.97	2.32	3.258(6)	162.6
N3-H3B...O6$^{\#2}$	0.97	2.41	3.273(6)	147.6
C12-H12...O5$^{\#1}$	0.98	2.57	3.517(7)	163.8
O10-H10E...O8	0.85	2.31	3.119(6)	159.1
O11-H11D...O4	0.85	2.08	2.816(6)	144.7
O10-H10D...O4	0.85(3)	2.19(9)	2.752(7)	123(9)
O10-H10D...O4	0.85(3)	2.19(9)	2.752(7)	123(9)

Complex (III)

N1-H1B...O1$^{\#1}$	0.85(3)	2.21(4)	3.039(4)	166(3)
N2-H2B...O2$^{\#2}$	0.83(3)	2.30(3)	3.066(4)	154(3)
N2-H2A...O5$^{\#2}$	0.87(3)	2.64(3)	3.088(5)	113(2)
C14-H14B...O6$^{\#1}$	0.98	2.43	3.374(5)	160.8
C12-H12A...O4$^{\#2}$	0.98	2.39	3.350(4)	165.8
C8-H8...O5	1.00	2.48	3.251(4)	133.5
C6-H6A...O6$^{\#3}$	0.99	2.52	3.502(4)	171.7
C2-H2C...O3	1.00	2.59	3.151(4)	115.6
O2-H2...O4	0.84	1.83	2.634(3)	159.0
O1-H1...O6	0.84	1.78	2.618(3)	174.0
N1-H1B...O1$^{\#1}$	0.85(3)	2.21(4)	3.039(4)	166(3)
N2-H2B...O2$^{\#2}$	0.83(3)	2.30(3)	3.066(4)	154(3)
N2-H2A...O5$^{\#2}$	0.87(3)	2.64(3)	3.088(5)	113(2)
C14-H14B...O6$^{\#1}$	0.98	2.43	3.374(5)	160.8
C12-H12A...O4$^{\#2}$	0.98	2.39	3.350(4)	165.8
C8-H8...O5	1.00	2.48	3.251(4)	133.5
C6-H6A...O6$^{\#3}$	0.99	2.52	3.502(4)	171.7
C2-H2C...O3	1.00	2.59	3.151(4)	115.6
O2-H2...O4	0.84	1.83	2.634(3)	159.0
O1-H1...O6	0.84	1.78	2.618(3)	174.0
O1-H1...O6	0.84	1.78	2.618(3)	174.0
O2-H2...O4	0.84	1.83	2.634(3)	159.0
C2-H2C...O3	1.00	2.59	3.151(4)	115.6
C6-H6A...O6$^{\#3}$	0.99	2.52	3.502(4)	171.7
C8-H8...O5	1.00	2.48	3.251(4)	133.5
C12-H12A...O4$^{\#2}$	0.98	2.39	3.350(4)	165.8
C14-H14B...O6$^{\#1}$	0.98	2.43	3.374(5)	160.8
N2-H2A...O5$^{\#2}$	0.87(3)	2.64(3)	3.088(5)	113(2)
N2-H2B...O2$^{\#2}$	0.83(3)	2.30(3)	3.066(4)	154(3)
N1-H1B...O1$^{\#1}$	0.85(3)	2.21(4)	3.039(4)	166(3)
O1-H1...O6	0.84	1.78	2.618(3)	174.0
O2-H2...O4	0.84	1.83	2.634(3)	159.0
C2-H2C...O3	1.00	2.59	3.151(4)	115.6
C6-H6A...O6$^{\#3}$	0.99	2.52	3.502(4)	171.7
C8-H8...O5	1.00	2.48	3.251(4)	133.5
C12-H12A...O4$^{\#2}$	0.98	2.39	3.350(4)	165.8
C14-H14B...O6$^{\#1}$	0.98	2.43	3.374(5)	160.8
N2-H2A...O5$^{\#2}$	0.87(3)	2.64(3)	3.088(5)	113(2)
N2-H2B...O2$^{\#2}$	0.83(3)	2.30(3)	3.066(4)	154(3)
N1-H1B...O1$^{\#1}$	0.85(3)	2.21(4)	3.039(4)	166(3)

Complex (IV)

N1-H1A...O4S$^{\#1}$	0.91	2.19	3.015(15)	150.8
N1-H1B...O2$^{\#2}$	0.91	1.98	2.880(14)	169.6

N2-H2A...O1$^{\#2}$	0.91	2.03	2.925(13)	166.5
N2-H2A...O1S	0.91	2.72	3.158(14)	110.6
O1-H1...O1A	0.84	1.73	2.429(14)	139.0
N1A-H1AA...O6S	0.91	2.31	3.02(3)	135.1
N2A-H2AB...O9S	0.91	2.72	3.16(4)	110.6
N2A-H2AC...O5S	0.91	2.26	3.16(3)	176.0
O2A-H2AD...O2	0.877(14)	2.05(15)	2.445(13)	106(12)
O9S-H9SB...O6S$^{\#3}$	0.87	2.38	3.15(4)	148.7
**Complex (V)**				
C24-H24B...O1$^{\#1}$	0.98	2.43	3.338(18)	153.4
C23-H23...Cl1$^{\#1}$	0.99	2.75	3.576(17)	141.9
C7-H7...Cl3$^{\#2}$	0.99	2.83	3.782(16)	161.8
N3-H3B...Cl2	0.98	2.77	3.567(13)	138.8
N3-H3B...Cl1	0.98	2.83	3.512(13)	127.4
N3-H3A...Cl2$^{\#3}$	0.98	2.43	3.338(12)	153.1
N2-H2B...Cl3$^{\#2}$	0.98	2.69	3.613(13)	156.2
N1-H1B...Cl1	0.98	2.46	3.295(13)	142.8
N1-H1A...Cl1$^{\#1}$	0.98	2.48	3.418(12)	160.4
O3-H3...Cl2$^{\#2}$	0.866(13)	2.49(9)	3.221(11)	142(12)
O1-H1...Cl2$^{\#1}$	0.83	2.53	3.281(12)	150.5
O1-H1...Cl2$^{\#1}$	0.83	2.53	3.281(12)	150.5
O3-H3...Cl2$^{\#2}$	0.866(13)	2.49(9)	3.221(11)	142(12)
N1-H1A...Cl1$^{\#1}$	0.98	2.48	3.418(12)	160.4
N1-H1B...Cl1	0.98	2.46	3.295(13)	142.8
N2-H2B...Cl3$^{\#2}$	0.98	2.69	3.613(13)	156.2
N3-H3A...Cl2$^{\#3}$	0.98	2.43	3.338(12)	153.1
N3-H3B...Cl1	0.98	2.83	3.512(13)	127.4
N3-H3B...Cl2	0.98	2.77	3.567(13)	138.8
C7-H7...Cl3$^{\#2}$	0.99	2.83	3.782(16)	161.8
C23-H23...Cl1$^{\#1}$	0.99	2.75	3.576(17)	141.9
C24-H24B...O1$^{\#1}$	0.98	2.43	3.338(18)	153.4
**Complex (VII)**				
N3-H3B...Cl2$^{\#1}$	0.97	2.63	3.456(8)	143.4
N3-H3A...Cl1$^{\#2}$	0.97	2.46	3.378(7)	157.3
N2-H2B...Cl2$^{\#1}$	0.97	2.51	3.361(7)	145.9
N2-H2A...Cl1	0.97	2.42	3.334(7)	157.2
N1-H1B...Cl2$^{\#1}$	0.97	2.40	3.268(7)	148.7
N1-H1A...Cl2	0.97	2.37	3.296(7)	159.9
O3-H3...Cl1	0.863(13)	2.56(8)	3.057(6)	118(7)
O2-H2...Cl2	0.867(13)	2.43(6)	3.058(6)	130(6)
O1-H1...Cl1$^{\#2}$	0.860(13)	2.53(7)	3.071(7)	122(7)
O1-H1...Cl1$^{\#2}$	0.860(13)	2.53(7)	3.071(7)	122(7)
O2-H2...Cl2	0.867(13)	2.43(6)	3.058(6)	130(6)
O3-H3...Cl1	0.863(13)	2.56(8)	3.057(6)	118(7)

N1-H1A...Cl2	0.97	2.37	3.296(7)	159.9
N1-H1B...Cl2[#1]	0.97	2.40	3.268(7)	148.7
N2-H2A...Cl1	0.97	2.42	3.334(7)	157.2
N2-H2B...Cl2[#1]	0.97	2.51	3.361(7)	145.9
N3-H3A...Cl1[#2]	0.97	2.46	3.378(7)	157.3
N3-H3B...Cl2[#1]	0.97	2.63	3.456(8)	143.4

Symmetry transformations used to generate equivalent atoms: For (I), #1 = x + 1/2, -y + 3/2, - z + 1; #2 = x + 1, y, z; #3 = x + 1/2, - y + 1/2, - z + 1. For (III), #1 = x + 1, y, z ; #2 = x - 1, y, z; #3 = x, y, z - 1. For (IV), #1 = x + 1/2, - y + 3/2, - z + 1; #2 = x - 1/2, - y + 3/2,-z+1, #3 = x + 1, y, z. For (V), #1 = - x + 2, y - 1/2, - z + 1; #2 = x, y - 1, z; #3 = - x + 1, y - 1/2, - z + 1. For (VII), #1 = x+1/2, -y + 3/2, - z + 1; #2 = -x + 1, y + 1/2, - z + 1/2.

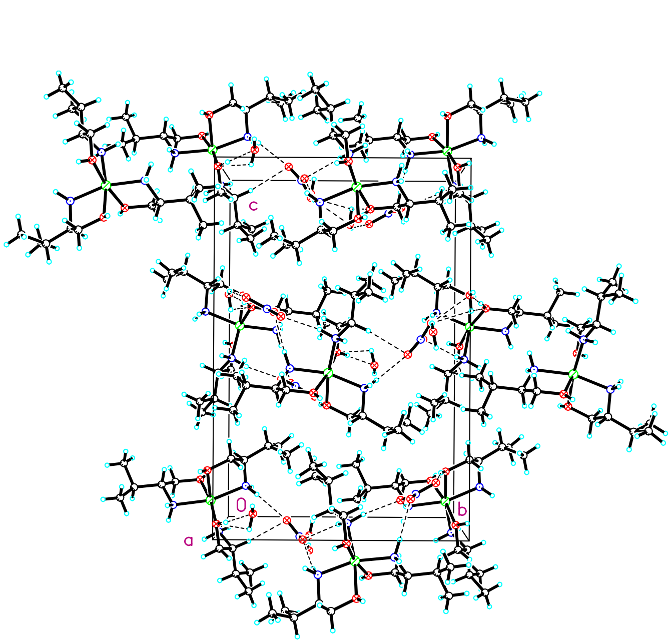

(a)

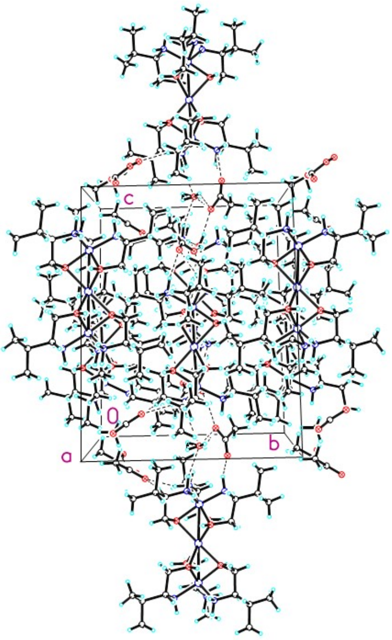

(b)

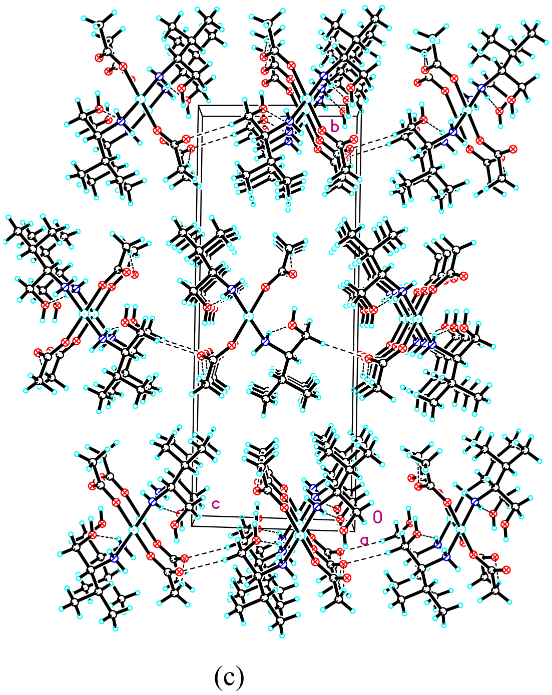

(c)

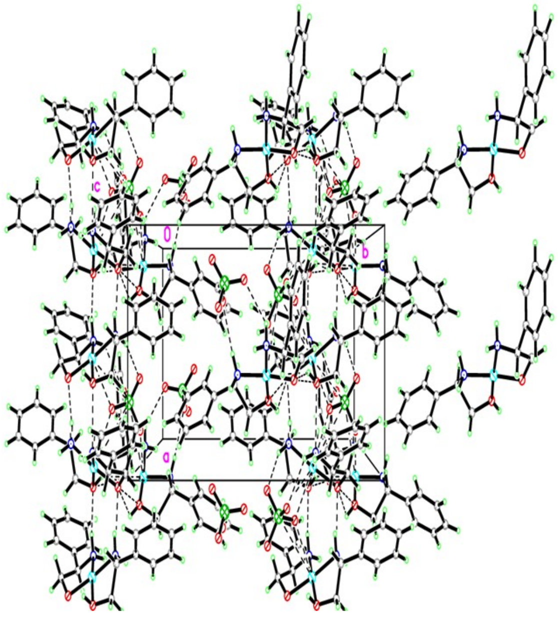

(d)

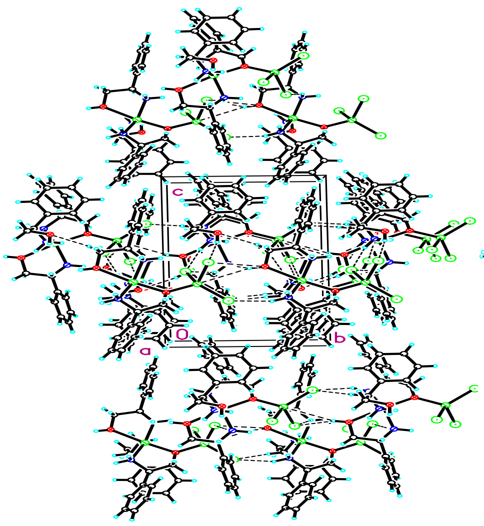

(e)

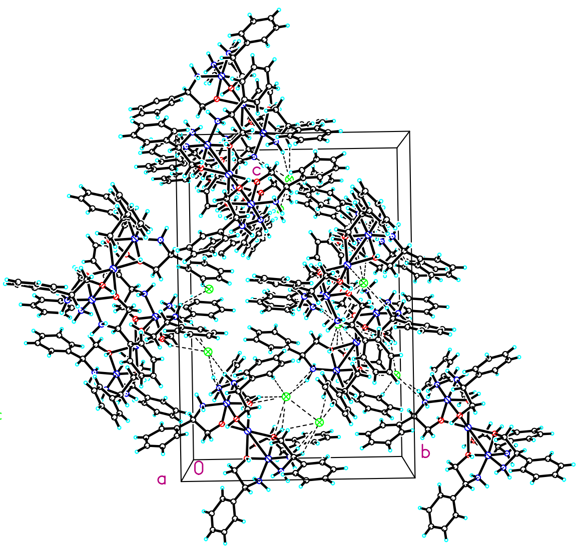

(f)

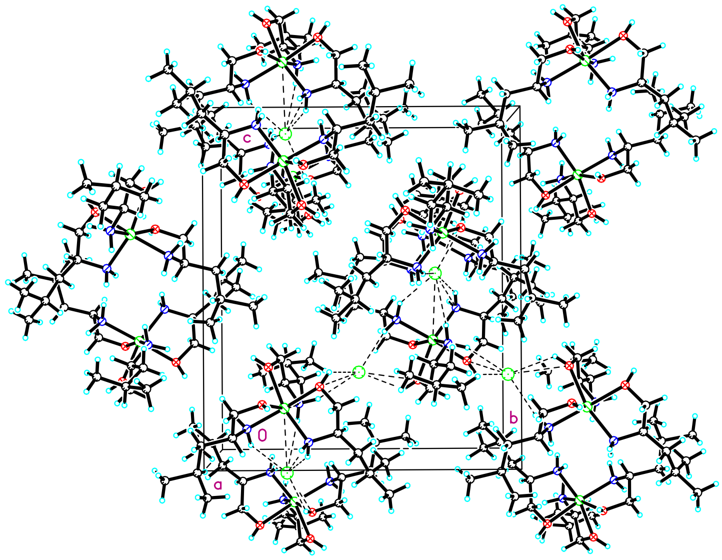

(g)

Figure S1 The crystal packing of complexes (I) – (VII), showing the formation of the three-dimensional network by hydrogen bonding interactions, (a) for (I), (b) for (II), (c) for (III), (d) for (IV), (e) for (V), (f) for (VI) and (g) for (VII), respectively.

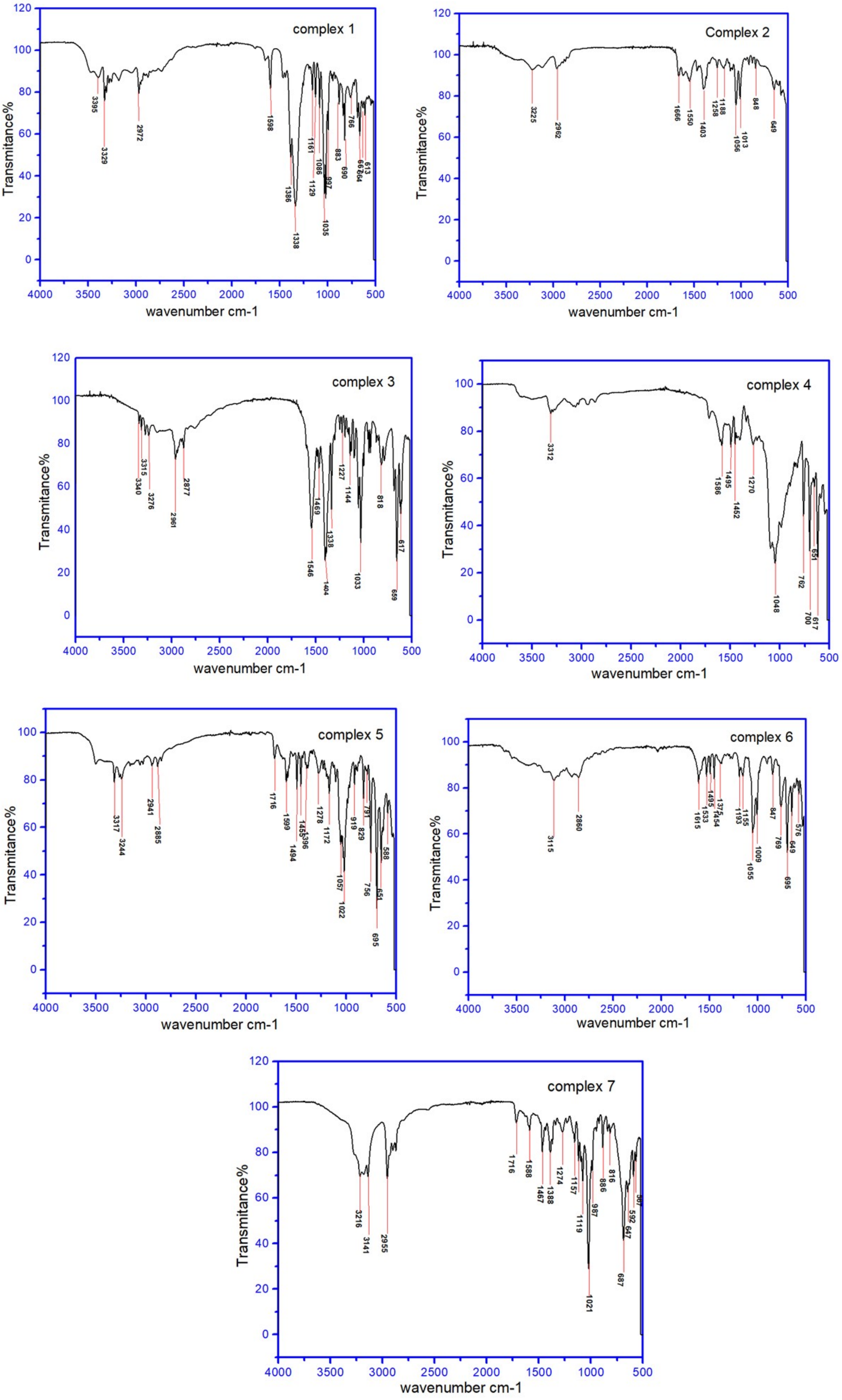

Figure S2 IR spectrums of complexes (I) - (VII).

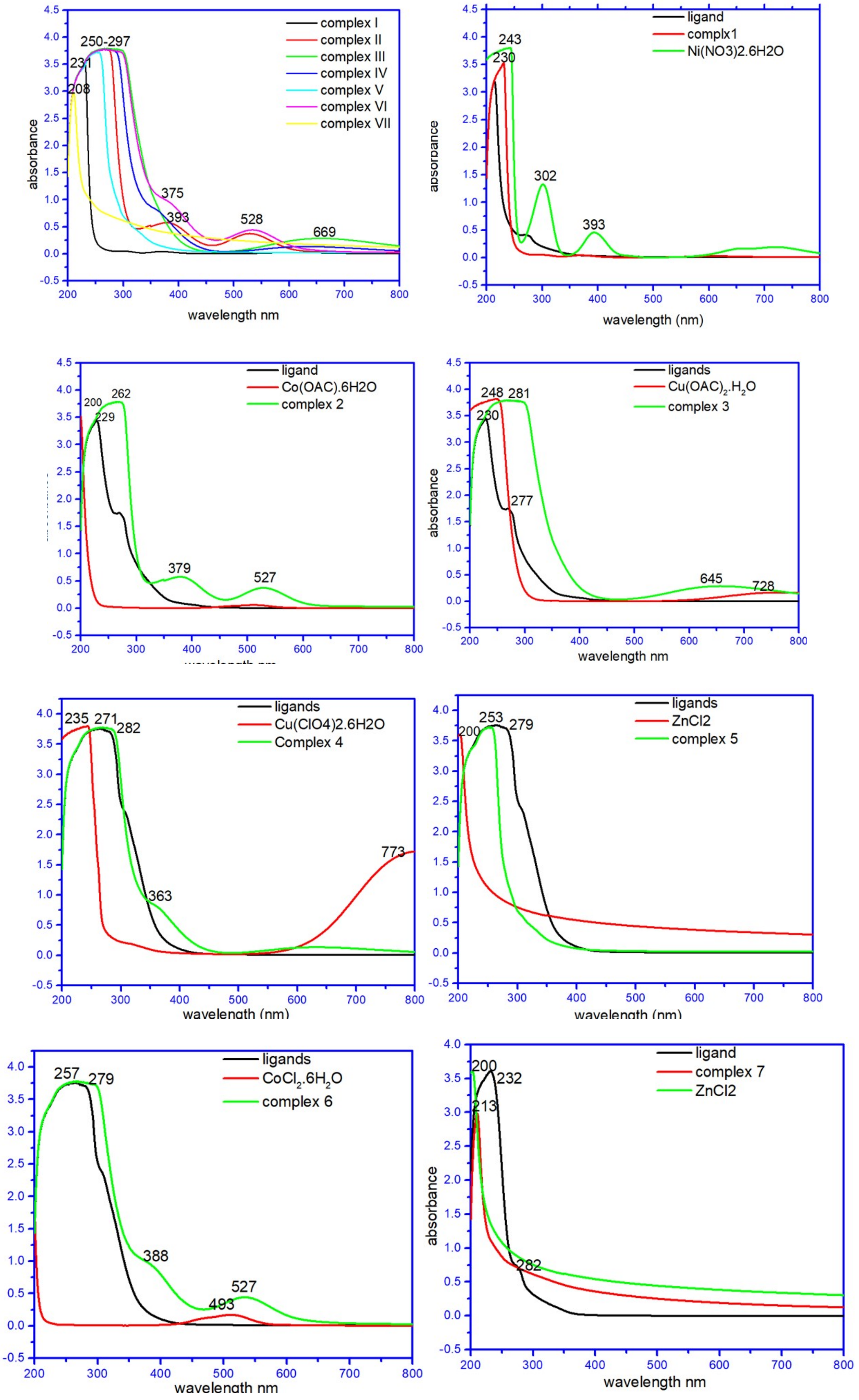

Figure S3 UV spectrum of complexes (I) - (VII).
